# Recent findings in the regulation of G6PD and its role in diseases

**DOI:** 10.3389/fphar.2022.932154

**Published:** 2022-08-24

**Authors:** Qingfei Meng, Yanghe Zhang, Shiming Hao, Huihui Sun, Bin Liu, Honglan Zhou, Yishu Wang, Zhi-Xiang Xu

**Affiliations:** ^1^ Key Laboratory of Pathobiology, Ministry of Education, Jilin University, Changchun, China; ^2^ Department of Urology, The First Hospital of Jilin University, Changchun, China; ^3^ School of Life Sciences, Henan University, Kaifeng, China

**Keywords:** glucose-6-phosphate dehydrogenase, pentose phosphate pathway, post-translational modifications, metabolic reprogramming, tumorigenesis

## Abstract

Glucose-6-phosphate dehydrogenase (G6PD) is the only rate-limiting enzyme in the pentose phosphate pathway (PPP). Rapidly proliferating cells require metabolites from PPP to synthesize ribonucleotides and maintain intracellular redox homeostasis. G6PD expression can be abnormally elevated in a variety of cancers. In addition, G6PD may act as a regulator of viral replication and vascular smooth muscle function. Therefore, G6PD-mediated activation of PPP may promote tumor and non-neoplastic disease progression. Recently, studies have identified post-translational modifications (PTMs) as an important mechanism for regulating G6PD function. Here, we provide a comprehensive review of various PTMs (e.g., phosphorylation, acetylation, glycosylation, ubiquitination, and glutarylation), which are identified in the regulation of G6PD structure, expression and enzymatic activity. In addition, we review signaling pathways that regulate G6PD and evaluate the role of oncogenic signals that lead to the reprogramming of PPP in tumor and non-neoplastic diseases as well as summarize the inhibitors that target G6PD.

## Introduction

Glucose-6-phosphate dehydrogenase (G6PD) is the only rate-limiting enzyme in the pentose phosphate pathway (PPP). PPP flow is therefore mainly regulated through G6PD expression or enzyme activity. PPP involves the formation of a bypass from glucose-6-phosphate, an intermediate product of glycolysis, which produces fructose-6-phosphate and glyceraldehyde-3-phosphate through two stages of oxidation and group transfer back to glycolysis, also referred to as the hexose monophosphate shunt.

PPP takes place in the cytoplasm and comprises oxidative (oxPPP) and nonoxidative (non-oxPPP) phases. In the oxidative phase, G6PD catalyzes glucose-6-phosphate to generate nicotinamide adenine dinucleotidephosphate (NADPH) and 6-phosphogluconolactone in an NADP^+^-dependent manner. NADPH is required for the synthesis of both intracellular fatty acids and cholesterol. It also scavenges reactive oxygen species (ROS) and maintains the reduction state of glutathione to combat oxidative stress. As a consequence, cells with a high demand for NADPH, such as tumor cells, exhibit a metabolic vulnerability that could be targeted by the inhibition of G6PD as a therapeutic strategy (Ju et al., 2020). Another important product of the non-oxPPP is ribose-5-phosphate (R5P), which provides important precursors for nucleotide synthesis. Rapidly proliferating cells require products to build cell blocks and maintain intracellular redox homeostasis (Rao et al., 2015). In addition, metabolites in the PPP can function as signaling molecules for the regulation of gene expression (Lin et al., 2015; Gao et al., 2019).

In this review, we focus on current findings in post-translational modifications (PTM) of G6PD and their roles in tumorigenesis and pathogenesis of non-neoplastic diseases.

## Transcriptional regulation of G6PD

### Transcription factors regulate G6PD expression

**FIGURE 1:**
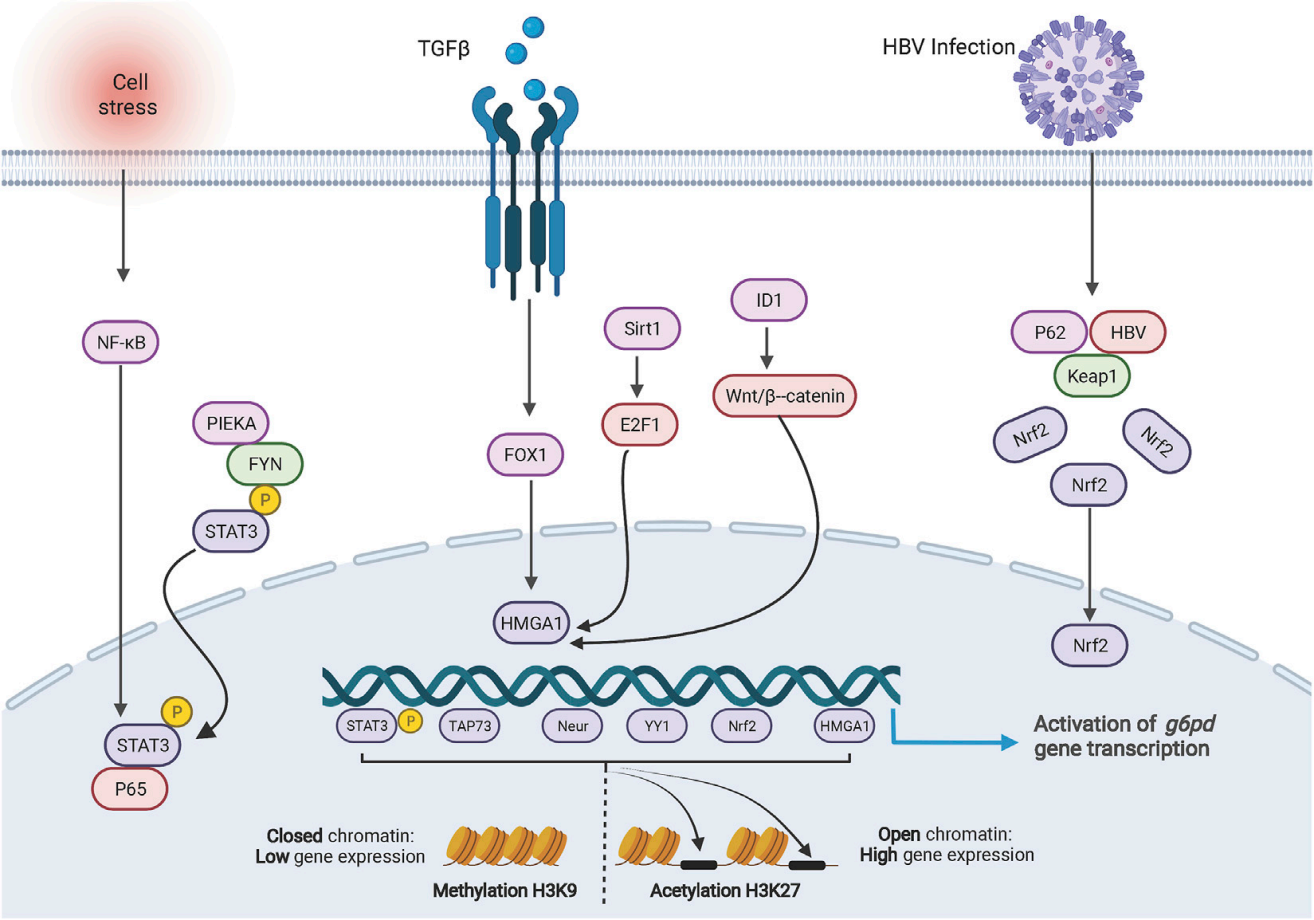
Transcriptional regulation of G6PD. The cartoon diagram on display consists of three main parts. On the left, activation of NF-ĸB in response to cellular stresses or the PIEKA-FYN complex leads to the phosphorylation and activation of STAT3, which results in the translocation of p-STAT3 to the nucleus and binding to the G6PD promoter enhancing transcription. In the middle section, signals regulate the expression of HMGA1 to promote G6PD transcription. On the right side, HBV protein forms a complex with intracellular protein p62 and KEAP1, resulting in translocation of NRF2 into the nucleus to promote G6PD expression. At the bottom, methylation and acetylation of histones are involved in transcriptional regulation of G6PD.

*G6PD* consists of 13 exons and 12 introns, which encode a product of 1,545 bp. The characterization of the promoter region shows 1) a high level (70%) of guanine and cytosine content; 2) a TATA box, which controls the accuracy and frequency of transcription initiation and is located in the -202 bp region upstream of the *G6PD* transcription start site (Gomez-Manzo et al., 2016). The promoter region of *G6PD* contains multiple binding sites for transcription factors. The transcription factors NeuroD1 (Li Z. et al., 2021), HMGA1 (Zhang R. et al., 2019; Gong et al., 2020), YY1 (Wu et al., 2018), c-MYC (Yin et al., 2017), p65 (Zhang et al., 2020), TAp73 (Du et al., 2013), Nrf2 (Liu et al., 2015; Zhang H.-S. et al., 2019; Lv et al., 2022), and pSTAT3 (Zhang et al., 2020; Sun M. et al., 2021) can directly and individually regulate *G6PD* transcription by binding to the *G6PD* promoter region (Figure 1). Additionally, dual transcription factors from the p65/pSTAT3 complex bind to the pSTAT3 binding site rather than the p65-binding site in the *G6PD* promoter region to stimulate *G6PD* transcription (Zhang et al., 2020).

### Transcriptional coactivators/repressors regulate G6PD expression

Transcriptional coactivators or corepressors are also involved in the regulation of *G6PD* transcription. Coactivators and repressors, which are cellular proteins that contain a DNA binding domain without directly binding to the promoter, assemble with transcription factors to form transcriptional complexes that enhance or repress gene transcription, respectively. In pancreatic ductal adenocarcinoma cells, the transcriptional coactivator yes-associated protein 1 interacts with TEA domain transcription factor 1 to regulate *G6PD* expression (Nie et al., 2021). In addition, HATs are involved in the regulation of transcription as coactivators. Acetylation of histones regulated by HATs loosens chromosome structure and facilitates the binding of DNA to transcription factors (Li W. et al., 2021). Histone deacetylase inhibitors (HDACs), NaBu, increases *G6PD* transcription by recruiting transcription factor Sp1 (Makarona et al., 2014). On the other hand, HDACs are transcriptional corepressors capable of transcriptional repression or silencing. For example, liver kinase B1 (LKB1)–AMP-activated protein kinase (AMPK) axis-mediated phosphorylation of histone deacetylase 10 (HDAC10) promotes its translocation to the nucleus to regulate *G6PD* expression (Shan et al., 2019).

### Non-coding RNA regulates the expression of G6PD

Small non-coding RNAs are also involved in the regulation of *G6PD* expression. Multiple microRNA binding sites exist in the 3′UTR region of *G6PD*. *MIR-206*, a skeletal muscle-specific microRNA, is a key regulator in skeletal muscle development. *MIR-206* functions pro-myogenically through direct binding of *G6PD* to restore differentiation of rhabdomyosarcoma cells (Coda et al., 2015). In addition, it can inhibit skeletal muscle cell proliferation by targeting *G6PD* (Jiang et al., 2019). MicroRNA has also been reported to inhibit tumor growth by targeting *G6PD*. In renal cell carcinoma, large-scale transcriptome and metabolic analyses showed that miR-146a-5p and miR-155-5p were involved in PPP reprogramming (Boguslawska et al., 2019). Furthermore, LINC00242 competitively bound miR-1-3p to free G6PD from miR-1-3p-mediated repression promoting gastric cancer progression (Deng et al., 2021).

## Post-Translational modification regulates G6PD expression in tumorigenesis

PTM of histones is an important epigenetic mechanism regulating the transcriptional activity of G6PD. Both acetylation and methylation modifications of histones have been identified as regulators of G6PD expression. Inhibition of histone deacetylase leads to the recruitment of transcription factor sp1 to the promoter region of G6PD (Makarona et al., 2014), which result in the increase in G6PD expression, suggesting that acetylation may be involved in the transcriptional regulation of G6PD. Recently, increased levels of H3K27Ac have been identified in the G6PD promoter region promoting HDAC10-driven transcription (Shan et al., 2019). Methylation modifications of histone lysine residues were also characterized as regulators of G6PD transcription. H3K9 methylation at G6PD promoter was significantly enriched, leading to the inhibition of G6PD expression (Lu et al., 2022). However, the specific lysine methyltransferases or demethylases that mediate histone methylation in G6PD transcription remains unclear.

In addition to regulating G6PD expression at the transcriptional level, PTMs are also involved in the stability of G6PD through the ubiquitin-proteasome system. Hypoxia activates G6PD expression, which could be reversed by ROS scavengers, suggesting that hypoxia may increase G6PD expression by inducing ROS accumulation. On the other hand, although G6PD expression is significantly reduced under hypoxic conditions and reversed by the proteasome inhibitor MG132, the specific mechanism remains unclear (Chettimada et al., 2015). Recently, von Hippel-Lindau (VHL) E3, an ubiquitin ligase, was found to be involved in the regulation of G6PD stability. VHL directly binds and ubiquitinates G6PD at the K366 and K403, which in turn degrades G6PD (Wang et al., 2019). In addition, SUMOylation and ubiquitination synergistically regulate the stability of G6PD. Silent information regulator 2 (Sirt2) directly binds to G6PD to increase enzyme activity through enhanced SUMOylation and inhibition of ubiquitination (Ni et al., 2021).

## Post-Translational modification of G6PD regulates enzyme activity in tumorigenesis

### G6PD phosphorylation

Phosphorylation modifications occur mainly on serine, tyrosine, and threonine residues, in which the hydroxyl group can be dehydrated with the phosphate group to form phosphate esters. Gu et determined, using mass spectrometry, that NF-κB-inducing kinase phosphorylation of G6PD at S40 enhances the enzymatic activity and promotes CD8^+^ effector T cells (Gu et al., 2021). Most reports have focused on the phosphorylation of G6PD tyrosine sites (Pan et al., 2009; Ma et al., 2021). G6PD is a substrate of the non-receptor tyrosine kinase family member Src. Several tyrosine sites of G6PD can be phosphorylated by Src, including Y112, Y428, and Y507. Among them, Y112 is considered to be the most important phosphorylation site of Src and phosphorylation at this site increases the enzymatic activity of G6PD and enhances PPP flow to promote tumorigenesis (Pan et al., 2009; Ma et al., 2021). Other members of the Src family can also directly bind phosphorylated G6PD. Fyn, a member of the SRC family, phosphorylates Y401 increasing the enzymatic activity of G6PD more than three-fold in erythrocytes (Mattè et al., 2020). In addition, salt-inducible kinase 3 (SIK3), a serine/threonine kinase, binds and phosphorylates G6PD at Y384 enhancing its enzymatic activity (Teesalu et al., 2017). Protein kinase A (PKA) inhibits the expression of SIK3 (Wang et al., 2011), which suggests that PKA and SIK3 may play opposing roles in the regulation of G6PD activity. This is consistent with previous reports that PKA inhibits G6PD enzyme activity (Xu et al., 2005). In addition to tyrosine and serine as potential phosphorylation sites for G6PD, G6PD is phosphorylated by polo-like kinase 1 at T406 and T466 sites increasing its enzymatic activity (Ma et al., 2017).

### G6PD O-linked GlcNAc

O-linked β-N-Acetylglucosamine (O-GlcNAc) is a reversible post-translational modification that occurs on serine or threonine residues. This process is regulated by the addition or removal of O-GlcNAc for O-GlcNAc transferase (OGT) and O-GlcNAcase (OGA), respectively (Zeng et al., 2016). Recent findings indicate that G6PD is dynamically O-GlcNAcylated at serine 84, which dramatically increases the enzymatic activity of G6PD. Meanwhile, G6PD glycosylation enhances PPP flow to the building blocks of macromolecular biosynthesis promoting the proliferation of tumor cells (Rao et al., 2015). Hypoxic or ERK-induced G6PD O-GlcNAcylation levels are increased in an OGT-dependent manner (Rao et al., 2015; Su et al., 2021). Thus, in addition to directly targeting the enzymatic activity of G6PD, targeting OGT may also be an effective strategy for inhibiting G6PD enzyme activity.

### G6PD acetylation

The level of acetylation of certain proteins in cells is determined by the balance between histone deacetylases (HDACs) and histone acetyltransferase (HATs), enzymes that add or remove acetyl groups from lysine residues, respectively (Li W. et al., 2021). KAT9/ELP3, an acetyltransferase, mediates G6PD K403 acetylation to inhibit the enzymatic activity of G6PD (Wang et al., 2014). Conversely, deacetylation of G6PD mediated by deacetylase Sirt2 enhances the enzymatic activity of G6PD and counteracts excessive oxidative stress (Wang et al., 2014; Xu et al., 2016). Furthermore, a report by Zhang et al. indicates that Sirt2 can bind to G6PD and regulate the deacetylation of G6PD K171 promoting the progression of hepatocellular carcinoma (Zhang et al., 2021). In addition to its role as a deacetylase involved in the regulation of G6PD enzyme activity, Sirt2 also maintains the stability of G6PD (Ni et al., 2021). Aspirin, a common clinical analgesic and antipyretic drug, has also been reported to be involved in the regulation of acetylation. It has been shown that aspirin inhibits tumor cell proliferation by inducing G6PD acetylation and correspondingly reducing the enzymatic activity of G6PD to increases oxidative stress (Raza et al., 2011; Ai et al., 2016).

### Newly identified post-translational modifications of G6PD

**FIGURE 2:**
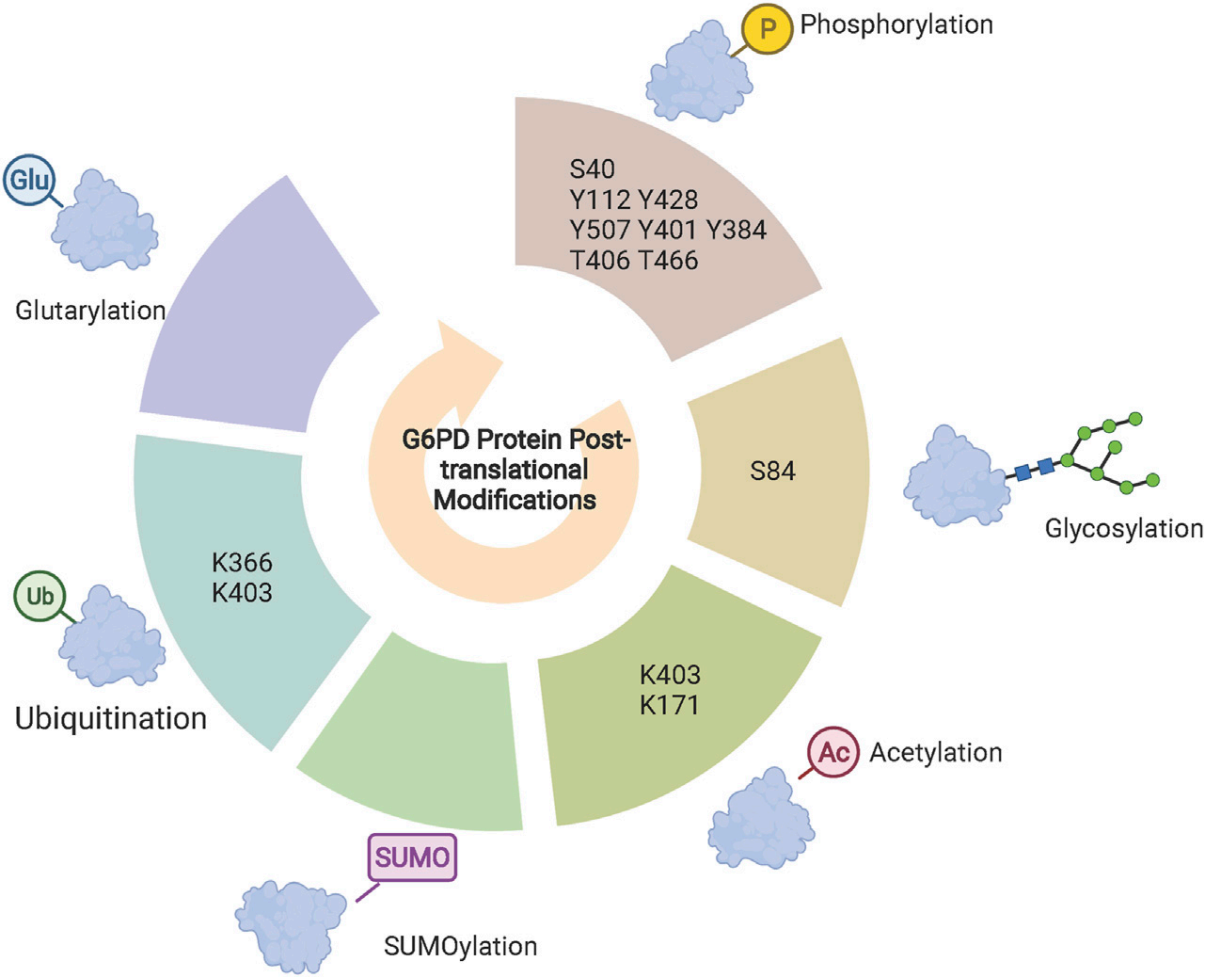
G6PD post-translational modifications. Phosphorylation, glycosylation, acetylation and glutarylation modifications regulate G6PD enzyme activity and specific sites identified are shown in the central circle. Ubiquitination and SUMOylation are synergistically involved in the regulation of G6PD protein stability. Acetylation and methylation of histones H3K27 and H3K9 regulate G6PD transcriptional expression, respectively.

Several novel post-translational modifications located on histone lysine residues have been identified including propionylation, butyrylation, 2-hydroxyisobutyrylation, succinylation, malonylation, glutarylation, crotonylation, and β-hydroxybutyrylation (Sabari et al., 2017). Notably, there are acylation modifications that are not exclusively restricted to histones. Deglutarylation of G6PD by deacylasesirtuin 5 increases its enzymatic activity (Zhou et al., 2016). Moreover, alterations in H4K8 2-hydroxyisobutyrylation can affect intracellular glucose metabolism (Huang et al., 2017), but whether G6PD is capable of 2-hydroxyisobutyrylation requires further investigation. On the other hand, the lactylation modification of histone lysine residues has been widely studied (Zhang D. et al., 2019). Existing studies have shown that P300 and HDAC1/3 act as lactylation modification “writers” or “erasers” to add or remove lactic acid groups on lysine residues of histones in macrophages, respectively (Zhang D. et al., 2019; Moreno-Yruela et al., 2022). Consistent with glutarylation modifications, lactylation modifications also occur in non-histone proteins. Glycolysis-derived lactate has been found to increase high mobility group box protein 1 lactylation to induce its ectopic transfer from the nucleus to the cytoplasm, enhancing its release from macrophages via exosomes (Yang et al., 2022). In conclusion, these newly identified post-translational modifications are not only restricted to histones (Sabari et al., 2017), but also other proteins (Yang et al., 2022), including G6PD (Zhou et al., 2016). Location and/or enzymatic activity of these targets are hence regulated through these post-translational modifications (Figure 2).

## Post-Translational modifications modify G6PD structure

The G6PD protein is composed of approximately 515 amino acid polypeptides and has an apparent molecular mass of approximately 59 kD. G6PD exist as an inactive monomer and active dimer as well as a tetramer (Hilf et al., 1975). Various factors, including pH value and ionic strength, affect the formation of dimers and tetramers. High values of pH and ion concentration promote the conversion of tetramers to dimers. Conversely, mild oxidative treatment results in the accumulation of tetramers with a corresponding decrease in dimers. Thus, there is an equilibrium between the dimers and tetramers (Hilf et al., 1975). In addition to factors regulating the structure of G6PD, NADPH converts dimers, but not tetramers, to monomers (Bonsignore et al., 1971). Therefore, NADPH is considered a potent inhibitor of G6PD. Depletion of NADP^+^, a G6PD coenzyme, results in the conversion of the G6PD dimers into monomers; reincubation of NADP^+^ with the dissociated protein restores dimer expression. This indicates that dimers and monomers can be reversibly converted into each other (Cancedda et al., 1973; Au et al., 1999).

**FIGURE 3:**
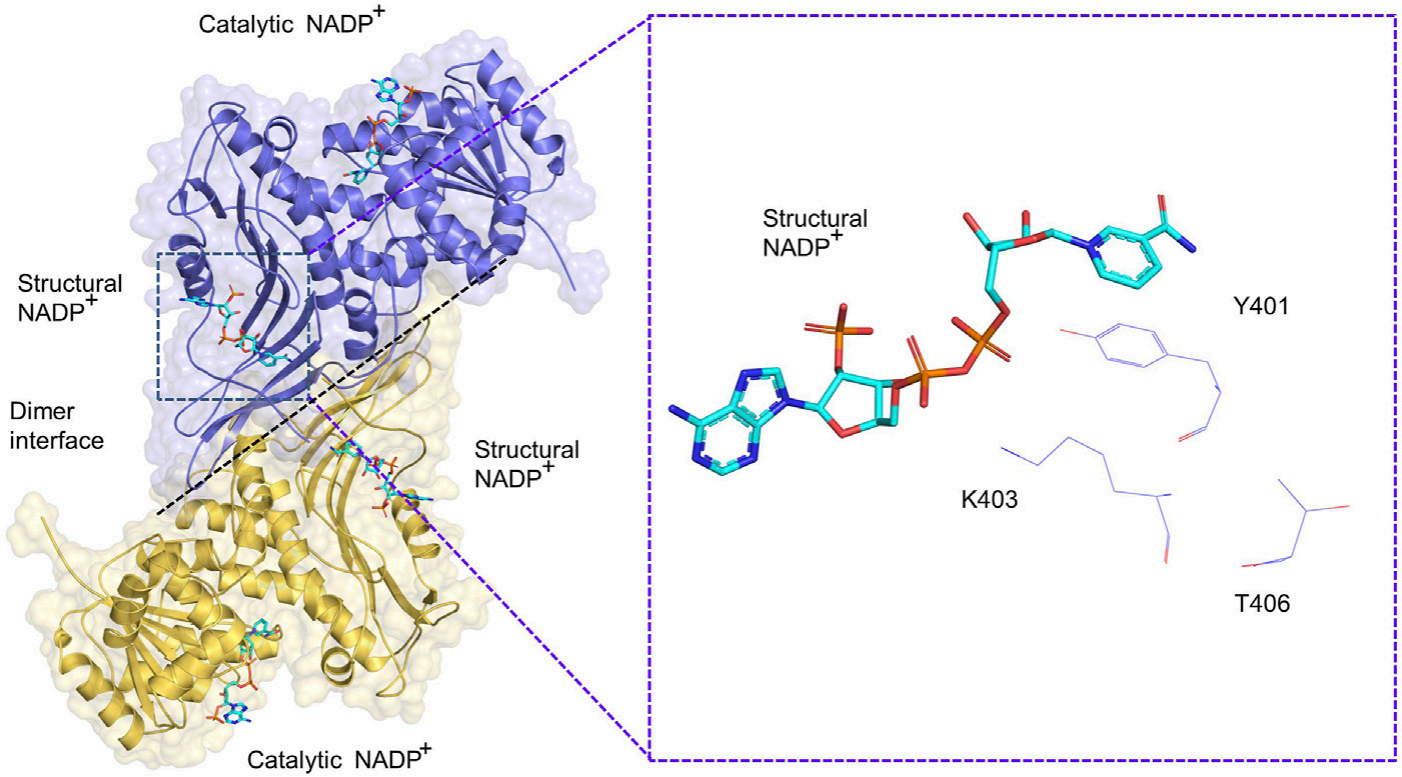
Schematic diagram of G6PD (PDB: 2BH9) dimer. A dimer consisting of two G6PD monomers, each of which includes a catalytic NADP^+^ and structural NADP^+^, respectively. The G6PD K403, Y401 and T406 sites are located close to the structural NADP^+^.

PTM modification of G6PD is involved in the regulation of dimerization. The G6PD molecule has two NADP^+^ binding sites including a structural NADP^+^ binding site and a coenzyme NADP^+^ binding site (Kotaka et al., 2005). Structural NADP^+^ sites are closer to the dimeric interface of G6PD than those of coenzyme G6PD sites, thus structural NADP^+^ binding sites are more important in regulating G6PD enzymatic activity and structural integrity than coenzyme structural sites (Au et al., 2000). In G6PD class I mutants, mutations located at the dimer interface and close to the NADP^+^ structural site lead to a 90% loss of function (Horikoshi et al., 2021), which further suggests that the NADP^+^ structural site is involved in the regulation of enzyme activity. A total of 57 amino acids have been identified at the dimer interface of G6PD, three of which are involved in dimer and monomer conversions, with the remaining sites in need of further investigation. In addition, mutations in T406, K403, and Y401 proteins, located at the dimer interface, promote the conversion of G6PD dimers to monomers. Specifically, FYN and Plk1 are directly phosphorylated to activate G6PD K401 and K406, promoting dimer formation and increasing enzyme activity, respectively (Ma et al., 2017; Mattè et al., 2020). In addition, KAT9-mediated acetylation of G6PD (K403) inhibits dimer formation of G6PD (Wang et al., 2014) (Figure 3).

## G6PD-Rrgulated downstream signalings

### G6PD inhibits ferroptosis

Ferroptosis is a novel type of iron-dependent regulated cell death (Dixon et al., 2012). Morphologically, ferroptosis is characterized by an increase in mitochondrial membrane density, reduction or disappearance of mitochondrial cristae, and rupture of the external mitochondrial membrane. Mechanistically, the accumulation of lipid peroxidation by the Fenton reaction between iron ions and ROS in cells leads to ferroptosis. NADPH is an important intracellular reducing equivalent to neutralize ROS and maintain redox homeostasis. According to the MetaCyc database (Caspi et al., 2020), there are at least 143 reactions for the conversion of NADP to NADPH, but only a limited number of these reactions are considered to be contributed significantly from NADP to NADPH conversion. The major source of NADPH in mammals is folate metabolism (methylenetetrahydrofolate dehydrogenase), glutaminolysis (malic enzymes), and oxPPP (G6PD, 6-Phosphogluconate dehydrogenase; 6PGD), of which G6PD is the largest contributor to NADPH production (Chen et al., 2019). Activation of PPP produces NADPH, which promotes resistance of clear cell renal cell carcinoma to ROS and ferroptosis (Zheng et al., 2021). In addition, it has been shown that the expression of cytochrome P450 oxidoreductase (POR), a positive regulator of ferroptosis, is significantly increased in G6PD knockdown hepatocellular carcinoma (HCC) cells, which suggests that G6PD may inhibit ferroptosis through POR (Cao et al., 2021). Thus, G6PD may regulate ferroptosis in an NADPH-dependent manner.

### G6PD-mediated metabolites regulate amp-activated protein kinase

Most studies have shown that alterations in signaling pathways can affect metabolites in PPP. Notably, G6PD-mediated metabolites can also regulate signaling molecules. G-6-phosphogluconolactone, a catalytic product of G6PD, can directly bind to Src to enhance the recruitment of protein phosphatase 2A and inhibit the activation of AMPK (Gao et al., 2019). In addition, Ru-5-P, the main metabolite of oxPPP, inactivates AMPK by inhibiting the formation of liver kinase B1 (Lin et al., 2015).

## Role of G6PD In Non-Neoplastic diseases

### G6PD and virus infection

Pathogen infections are more likely to occur in G6PD-deficient subjects because they have a decreased ability to activate the innate immune response (Yen et al., 2020). The Zika virus (ZIKV) genome is made up of a single-strand, positive-sense RNA with only 10 genes bordered by two untranslated sections (Savidis et al., 2016). ZIKV infection elicits a glycolytic response, as shown by increased extracellular acidification rate and expression of key glycolytic genes (*GLUT1*, *HK2*, *TPI*, and *MCT4*), according to bioinformation studies (Tiwari et al., 2017; Singh et al., 2020). Furthermore, infection with ZIKV leads to metabolic reprogramming and diversion of glycolytic carbon to PPP (Yau et al., 2021). Therefore, it suggest that ZIKV may increase the flow of PPP by upregulating enzymes including G6PD. In addition, it has been shown that activation of AMPK, a switch in energy metabolism, attenuates ZIKV infection of host cells (Singh et al., 2020). Indeed, pharmacological inhibition or knockdown of AMPK reduces G6PD expression (Shan et al., 2019). Thus, a potential regulatory mechanism for ZIKV virus infection of host cells may be mediated through the AMPK-G6PD axis. Similarly, during Kaposi’s sarcoma-associated herpesvirus (KSHV) infection of the human dermal microvascular endothelial, the metabolic pathway shifts from glycolysis to PPP, which is accompanied by a KSHV-induced increase in G6PD and transketolase expression (Sriram et al., 2008). The enhancement in PPP provides KSHV with a supply of nucleotides for the synthesis of host genes necessary for infection or for the synthesis of viral genes during early cellular bursts of the virus. Conversely, it is worth noting that the influenza virus reduces *G6PD* expression and enzyme activity, leading to an increase in oxidative stress and virus replication (De Angelis et al., 2021). Consistent with influenza virus infection, HIV, influenza A, respiratory syncytial virus, and enterovirus 71 induce oxidative stress and are usually suppressed by antioxidants like N-acetyl cysteine (Jain et al., 2020). In conclusion, the above studies that G6PD plays different roles in different types of viral infections.

Since 2020, the coronavirus disease (COVID-19) was declared as global pandemic, with hundreds of millions of people infected worldwide and increasing numbers of people becoming infected to date. However, no specific antiviral medications are currently available. There have been clinical trials using chloroquine and hydroxychloroquine (CQ/HCQ) to treat COVID-19. Several studies have shown that COVID-19 patients with G6PD deficiency show severe hemolysis during treatment with CQ/HCQ, which increase intracellular ROS in therapeutic dosages (da Rocha et al., 2021). Therefore, it is necessary to check the G6PD status of patients if CQ/HCQ is used to treat COVID-19.

Virus induces global changes of PTMs in host cell during infection to facilitate its successful infection and dissemination (Hu et al., 2020). To generate progeny virus, influenza virus replication requires a substantial number of nucleic acids for the synthesis of viral RNA (vRNA), complementary RNA (cRNA), and messenger RNA (mRNA). Vast amounts of energy are also required in the process of generating large amounts of RNAs. Pyruvate kinase M2 (PKM2), which catalyzes the production of ATP in glycolysis, becomes more acidic due to increased phosphorylation after influenza virus infection, and phosphorylated PKM2, which is active as a protein kinase, binds to RNA-dependent RNA polymerase involved in vRNA replication. Therefore, inhibition of PKM2 may be an effective strategy to attenuate viral replication. Whether G6PD phosphorylation is activated after infection to promote viral replication still needs further study (Miyake et al., 2017).

### G6PD and vascular diseases

Vascular remodeling is an important pathological phenotypic change in cardiovascular diseases, including hypertension and atherosclerosis, in which vascular smooth muscle plays an important role (Gong et al., 2021). Vascular smooth muscle cells (SMCs) undergo several alterations during biological processes, including phenotypic transformation, proliferation, and apoptosis during disease progression. Multiple studies have shown that G6PD deficiency increases the risk of cardiovascular disease, which implies that G6PD may act as a regulator of SMCs (Pes et al., 2019; Parsanathan and Jain, 2020). Differentiated SMCs located in the middle layer of the vessel wall can contract and relax to regulate blood flow through the circulatory system. SMCs-restricted gene (*Myocd*, *Tagln*, *Myh11*, and *Cnn1*) expression maintains SMCs in a differentiated state; in contrast, downregulation of SMCs-restricted gene expression leads to SMCs cell dedifferentiation causing vascular remodeling. Pharmacological inhibition of G6PD or knockdown of G6PD promotes SMCs-restricted gene expression to maintain vascular function (Dhagia et al., 2021). Therefore, G6PD maintains the dedifferentiated state of SMCs cells to avoid impaired vascular function. In addition, G6PD regulates the relaxation and contraction of vascular smooth muscle by altering the opening and closure of ion channels. G6PD can be activated by protein kinase C to elicit intracellular free Ca^2+^ and thus enhance the contraction of vascular smooth muscle (Ata et al., 2011). Conversely, pharmacological inhibition of G6PD relaxes vascular smooth muscle by opening potassium channels (Farrukh et al., 1998). G6PD-mediated metabolites are also involved in the regulation of vascular smooth muscle contraction. NADPH, the metabolite catalyzed by G6PD, relaxes vascular smooth muscle by inhibiting the dimer formation of PKG1α (Neo et al., 2013; Patel et al., 2014).

## Inhibitors

Small molecule inhibitors are useful tools for studying the function of metabolic enzymes. To date, there are 265 compounds that could be potential G6PD inhibitors according to data from BRENDA (https://www.brenda-enzymes.org). However, no details of the specific inhibitors of G6PD are yet available. In the following section, we review the G6PD inhibitors that are widely used in basic research and summarize their concentration and duration of application in different cells and animal models (Tables 1, 2).

**TABLE 1:** The effective dosages and durations or the application of G6PD inhibitors in cancer cells.

Inhibitors	Cell lines	Cancer type	Dose (μM)	Duration (H)	References
6-An	H1944	Lung cancer	56.37 ± 2.93	48	Sun et al., (2022)
	H1299	Lung cancer	202.40 ± 39.21	48	Sun et al., (2022)
	H1975	Lung cancer	6.91 ± 0.77	48	Sun et al., (2022)
	A549	Lung cancer	56.27 ± 2.72	48	Sun et al., (2022)
	A549/H460/H358/H441	Lung cancer	62.5	72	Best et al., (2019)
	A549	Lung cancer	500	18–24	Budihardjo et al., (1998)
	T98G	Brain glioblastoma	250	18–24	Budihardjo et al., (1998)
	MCF-7	Breast cancer	125	18–24	Budihardjo et al., (1998)
	OVCAR	Ovarian cancer	31	18–24	Budihardjo et al., (1998)
	U251	Brain glioblastoma	1,000	-	Sun et al., (2021b)
	786-O	kidney cancer	1,000	24	Zhang et al., (2020)
	PC3	Prostate cancer	100	24	Whitburn et al., (2022)
	LNCaP	Prostate cancer	100	24	Whitburn et al., (2022)
	MOLM-14/OCI-AML2/L60/OCI-AML3	-	100	48	Poulain et al., (2017)
	VSMCs	-	1,000	12	Dong et al., (2015)
	HEAC	-	100	12	Dong et al., (2015)
	PASM	-	1,000	72	Chettimada et al., (2015)
	Rat/Mouse neuronglia	-	10	24	Tu et al., (2019)
	Primary hepatocytes cell	-	5,000	0.2	Gupte et al., (2009)
DHEA	231-C3/231-M1	Breast cancer	200	12	Luo et al., (2022)
	HeLa	Cervical cancer	200	0.1	Roshanzadeh et al., (2019)
	WSU - HN6	Oral carcinoma	50	-	Wang et al., (2020)
	CAL27	Tongue carcinoma	50	-	Wang et al., (2020)
	GM00558	-	100	0.2	Cosentino et al., (2011)
	Human red blood cells	-	200	24	Handala et al., (2017)
	MEF	-	100	7	Heiss et al., (2013)
	Rat/Mouse neuronglia	-	100	24	Tu et al., (2019)
	Primary hepatocytes cell	-	100	10	Gupte et al., (2009)
	Pulmonary artery smoot muscle cell	-	100	72	Chettimada et al. (2015)
	Human aortic endothelial cell	-	100	12	Parsanathan and Jain, (2020)
Polydatin	HESCC	Esophageal carcinoma	100–300	24	Su et al., (2021)
	MCF-7	Breast cancer	30	24	Mele et al., (2019)
	HNSCC	Head and neck squamous cell carcinoma	22	24	Mele et al., (2018)
	HNSCC	Head and neck squamous cell carcinoma	17	48	Mele et al., (2018)
NEOU	H446	Lung cancer	10	48	Wang et al., (2022)
	SMCs	-	1	48	Dhagia et al., (2021)
Epi	A7r5	-	50	24	Dhagia et al., (2021)
DP20	Primary bone marrow cells	-	0.9	24	Hashimoto et al., (2020)

**TABLE 2:** The effective dosages and therapeutic durations of G6PD inhibitors in animal models of cancer.

Inhibitors	Organism	Dose	Duration	Injection type	References
6-An	Mouse	4 mg/kg/3d	-	Intraperitoneal injection	Sun et al., (2021b)
	Mouse	23 mg/kg/d	-	Intraperitoneal injection	Zhang et al., (2020)
	Mouse	20 mg/kg/10d	40d	Intraperitoneal injection	Best et al., (2019)
	Mouse	5 mg/kg/day	23d	Intraperitoneal injection	Poulain et al., (2017)
DHEA	Mouse	80 mg/kg/3d	20d	Intraperitoneal injection	Wang et al., (2020)
NEOU	Mouse	1.5 mg/kg/d	21d	Intraperitoneal injection	Kitagawa et al., (2021)
	Mouse	1.5 mg/kg/d	28d	Intraperitoneal injection	Joshi et al., (2020)
Epi	Rats	30 mg/kg/d	28d	Intraperitoneal injection	Dhagia et al., (2021)
Polydatin	Mouse	5 mg/kg/d	14d	Intraperitoneal injection	Su et al., (2021)
	Mouse	100 mg/kg	-	Intraperitoneal injection	Mele et al., (2018)

Dehydroepiandrosterone (DHEA) was identified as a non-competitive G6PD inhibitor in 1960 (Marks and Banks, 1960). DHEA sulfate (DHEAs) is an androgen produced by the adrenal glands. Humans have the highest levels of circulating DHEAs of all the primates with levels that are generally higher in males (3,200 ng/ml) than those in females (2000 ng/ml) (Nyce, 2021). DHEAs is an ineffective inhibitor of G6PD and is only transported into cells via organic anion transport protein (OATP), which is subsequently desulfated by sulfate esterase (SS) to eventually produce DHEA that inhibits G6PD activity (Klinge et al., 2018). Compared with hydrophilic DHEAs, lipophilic DHEA can function freely across cell membranes. Therefore, DHEA is widely used in cancer research to block G6PD enzyme activity and inhibit the proliferation and migration of cancer cells (Wang et al., 2020; Luo et al., 2022). Moreover, DHEA decreases intracellular NADPH levels by inhibiting G6PD, of which the effect is more pronounced under glucose deprivation (Roshanzadeh et al., 2019). However, Ghergurovich et al. showed that DHEA inhibited the enzymatic activity of G6PD in HepG2 cells, but this effect was not sustained (Ghergurovich et al., 2020). In addition to its role in cancer therapy, DHEA can be potentially beneficial in the treatment of pulmonary hypertension and protecting against ribavirin antiviral therapy-induced hemolysis (Patel et al., 2014; Handala et al., 2017). Some men in the United States take oral DHEA to boost their androgen levels to prevent aging, but no scientific proof has been obtained. DHEA can significantly inhibit G6PD enzyme activity, resulting in increased susceptibility to COVID-19 (Nyce, 2021).

6-Aminonicotinamide (6-An) is a competitive non-specific G6PD inhibitor that competitively binds to NADP^+^, to inhibit G6PD enzyme activity (Köhler et al., 1970). G6PD and 6PGD can generate NADPH from NADP^+^, which suggests that 6-An can also bind competitively with 6PGD to inhibit its activity during oxPPP. The concentrations of 6-An thus should be considered when it is used to inhibit G6PD enzyme activity. 6-An does not affect G6PD, but instead, blocks 6PGD(Aurora et al., 2022). Earlier *in vivo* studies revealed that 6-An inhibits the carbon-atom transfer from glucose to ribose and suppresses oxPPP (Köhler et al., 1970). In addition, 6-An selectively enhances the toxicity of cisplatin, melphalan, and nitrogen mustard to promote apoptosis of tumor cells *in vitro* (Budihardjo et al., 1998).

Additional drugs have been identified to inhibit the enzymatic activity of G6PD. Polydatin, an active ingredient extracted from the traditional Chinese medicine *Polygonum multiflorum*, was identified to inhibit the activity of G6PD enzymes and NADPH in a dose-dependent manner thus suppress the growth and metastasis of tumor cells (Mele et al., 2018). Additionally, (N-ethyl-N = -[(3β,5α)-17-oxoandrostan-3-yl]urea, NEOU) has been reported to inhibit G6PD activity (Joshi et al., 2020).

## Summary and perspectives

G6PD is the rate-limiting enzyme of the PPP. Along with serving as biosynthetic substrates, the G6PD-mediated metabolitesRu-5-P and NADPH regulate downstream signaling cascades and induce tumorigenesis (Lin et al., 2015). Lactatemay be employed as a substrate for lactylation modifications to regulate the expression of downstream genes. Lactylation modifications of non-histone proteins may be of great interest for future research, even if no relevant reports are currently available (Sun L. et al., 2021).

In addition, we reviewed the role of G6PD in tumorigenesis and related non-neoplastic diseases, of which we mainly focused on the role of post-translational modifications of G6PD. Post-translational modifications of histones, transcription factors, and other upstream multiple signals are involved in regulating the expression of G6PD. Glycosylation and phosphorylation modifications of G6PD promote dimer formation and increase enzyme activity (Rao et al., 2015; Zeng et al., 2016; Ma et al., 2017). Conversely, acetylation modifications promote dimer to monomer conversion and inhibit enzyme activity G6PD (Wang et al., 2014; Zhang et al., 2021). G6PD not only plays a role in tumorigenesis, but also in the process of viral infection. Briefly, viruses may inhibit intracellular metabolism and reduce the enzymatic activity of G6PD to promote viral infection during the early stages. Furthermore, viruses may activate metabolic pathways, including PPP, to promote viral replication at later stages. Finally, inhibitors of G6PD were summarized and the potential of G6PD as a clinical therapeutic target was evaluated.

Multiple post-translational modification sites of G6PD were identified by mass spectrometry. Serine at position 84 of G6PD could be glycosylated to increase the enzyme activity (Rao et al., 2015). In contrast, the enzyme activity was abolished by acetylation modification of lysine at 403. However, the reasons that changes in modifications affect enzyme activity need further investigation. In addition, serine is widely known to be phosphorylation modified, but no phosphorylation modification was identified at serine 84. Although a variety of G6PD modifications have been identified, there are still many questions that deserve further investigation. Based on this review, two questions were subsequently raised 1) Is there a prior order of post-translational modifications that occur in G6PD? 2) How do the various post-translational modifications collaborate? In summary, we highlight the role of post-translational modifications of G6PD in regulating structure, enzyme activity, and function. Therefore, targeting post-translational modifications of G6PD may serve as a novel therapeutic strategy.

## Abbreviations

cRNA, complementary RNA; G6PD, Glucose-6-phosphate dehydrogenase; HATs, Histone acetyltransferase; HDACs, Histone deacetylases; mRNA, messenger RNA; NADPH, Nicotinamide adenine dinucleotide phosphate; non‐oxPPP, Nonoxidative pentose phosphate pathway; OGA, O‐GlcNAcase; O‐GlcNAc, O‐linked β‐N‐Acetylglucosamine; OGT, O‐GlcNAc transferase; Oxppp, Oxidative pentose phosphate pathway; PKA, protein kinase A; PPP, Pentose phosphate pathway; PTM, post-translational modifications; R5P, Ribose-5-phosphate; ROS, Reactive oxygen species; R5P, Ribose-5‐phosphate; SIK3, salt‐inducible kinase 3; Sirt2, Silent information regulator 2; SMCs, Vascular smooth muscle cells; VHL, von Hippel-Lindau; vRNA, viral RNA; ZIKV, Zika virus.
